# Developmental pathways inferred from modularity, morphological integration and fluctuating asymmetry patterns in the human face

**DOI:** 10.1038/s41598-018-19324-y

**Published:** 2018-01-17

**Authors:** Mirsha Quinto-Sánchez, Francesc Muñoz-Muñoz, Jorge Gomez-Valdes, Celia Cintas, Pablo Navarro, Caio Cesar Silva de Cerqueira, Carolina Paschetta, Soledad de Azevedo, Virginia Ramallo, Victor Acuña-Alonzo, Kaustubh Adhikari, Macarena Fuentes-Guajardo, Tábita Hünemeier, Paola Everardo, Francisco de Avila, Claudia Jaramillo, Williams Arias, Carla Gallo, Giovani Poletti, Gabriel Bedoya, Maria Cátira Bortolini, Samuel Canizales-Quinteros, Francisco Rothhammer, Javier Rosique, Andres Ruiz-Linares, Rolando Gonzalez-Jose

**Affiliations:** 1 Ciencia Forense, Facultad de Medicina, Universidad Nacional Autónoma de México, Ciudad de México, Mexico; 2 Instituto Patagónico de Ciencias Sociales y Humanas. Centro Nacional Patagónico, CONICET, Puerto Madryn, Argentina; 3 Departament de Biologia Animal, de Biologia Vegetal i d’Ecologia, Facultat de Biociències, Universitat Autònoma de Barcelona, Avinguda de l’Eix Central, Edifici C, E-08193 Bellaterra (Cerdanyola del Vallès), Spain; 4 Posgrado en Antropología Física, Escuela Nacional de Antropología e Historia, Ciudad de México, Mexico; 5 Superintendência da Polícia Técnico-Científica do Estado de São Paulo. Equipe de Perícias Criminalísticas de Ourinhos, São Paulo, Brazil; 6 Department of Genetics, Evolution and Environment, and UCL Genetics Institute, University College London, London, UK; 7 Licenciatura en Antropología Física, Escuela Nacional de Antropología e Historia, Ciudad de México, Mexico; 8 Departamento de Tecnología Médica, Facultad de Ciencias de la Salud, Universidad de Tarapacá, Arica, Chile; 9 Departamento de Genética e Biologia Evolutiva, Instituto de Biociências, Universidade de São Paulo, São Paulo, Brazil; 10 Posgrado en Antropología, Instituto de Investigaciones Antropológicas, Universidad Nacional Autónoma de México, Ciudad de México, Mexico; 11 Universidad de Antioquia, Medellín, Colombia; 12 Laboratorios de Investigación y Desarrollo, Facultad de Ciencias y Filosofía, Universidad Peruana Cayetano Heredia, Lima, Peru; 13 Departamento de Genética, Instituto de Biociências, Universidade Federal do Rio Grande do Sul, Porto Alegre, Brazil; 14 Facultad de Química, UNAM, Mexico City, Mexico; 15 Facultad Instituto de Alta Investigación Universidad de Tarapacá, Programa de Genética Humana ICBM Facultad de Medicina Universidad de Chile y Centro de Investigaciones del Hombre en el Desierto, Arica, Chile; 16 Departamento de Antropología, Facultad de Ciencias Sociales y Humanas. Universidad de Antioquia, Medellín, Colombia; 17 MOE Key Laboratory of Contemporary Anthropology, Fudan University, Shanghai, China; 18 Aix Marseille Univ, CNRS, EFS, ADES, Marseille, France

## Abstract

Facial asymmetries are usually measured and interpreted as proxies to developmental noise. However, analyses focused on its developmental and genetic architecture are scarce. To advance on this topic, studies based on a comprehensive and simultaneous analysis of modularity, morphological integration and facial asymmetries including both phenotypic and genomic information are needed. Here we explore several modularity hypotheses on a sample of Latin American mestizos, in order to test if modularity and integration patterns differ across several genomic ancestry backgrounds. To do so, 4104 individuals were analyzed using 3D photogrammetry reconstructions and a set of 34 facial landmarks placed on each individual. We found a pattern of modularity and integration that is conserved across sub-samples differing in their genomic ancestry background. Specifically, a signal of modularity based on functional demands and organization of the face is regularly observed across the whole sample. Our results shed more light on previous evidence obtained from Genome Wide Association Studies performed on the same samples, indicating the action of different genomic regions contributing to the expression of the nose and mouth facial phenotypes. Our results also indicate that large samples including phenotypic and genomic metadata enable a better understanding of the developmental and genetic architecture of craniofacial phenotypes.

## Introduction

Modularity is an important principle of organization in biological systems, which is also manifest at the morphological level^1^. Modularity means that, on a complex structure, some set of traits exhibit greater internal integration in relation to the integration with traits belonging to other modules^2,3^. Modules observed in complex structures, such as the human face, consequently exhibit some degree of independence, according to their development, evolutionary, population-specific or experimentally induced conditions^4^.

The human face is a bilateral complex phenotype composed of a combination of structures that vary simultaneously in an integrated and modular way. For example, it has been reported that as part of the skull, the face is constrained in some way by variations in the skull base^5–9^. Conversely, other authors have suggested that it is closely linked to changes occurring in the neurocranium during brain growth^10^. This link has been evidenced in several experiments made on mouse models. Martínez-Abadías *et al*.^11^, for instance, analyzed integration of brain and skull in newborn mouse models of Apert syndromes and demonstrated that skull dysmorphology affects not only the coronal suture closure, but also the facial skeleton. The authors proposed that shared phenogenetic processes, such as regional differentiation by dynamic inductive signaling and repetitive patterning by quantitative interactions affecting several tissues during head morphogenesis, underlie not only premature suture closure, but additional cranial anomalies in Apert syndrome, including facial traits. Hallgrímsson *et al*.^12–14^ analyzed perturbations to craniofacial development in mouse models to disentangle the processes that determine covariation structure. These studies have shown that mutations that influence the growth of the chondrocranium produce a common pattern of integrated shape change throughout the mouse skull. Furthermore, Martínez-Abadías *et al*.^15,16^ have shown that this same integrated pattern is also a main component of covariation structure in the human skull. In other words, shape changes observed in the integrated effects of variation in chondrocranial growth in mouse mutants also correspond to a significant axis of covariation in humans.

Viewed from a different perspective, an analysis of simulated amniote developmental facial morphospaces suggest that epigenetic factors such as organismal geometry and shape impact facial morphogenesis and alter the locus of adaptive selection to variation in later developmental events^17^. More generally, the face can be represented after the Palimpsest Model^13,14^, that propose that patterns of covariation observed in the adult phenotype emerge from different processes of variance generation, that gradually overlap, add or integrate sequentially during the individual’s ontogeny^18^. Such particularities, then, define the face as a complex phenotype. However, the modular nature of structures into the face has been scarcely analyzed, to the exception of some recent approaches to the craniofacial modular patterns using network theory^19^.

Modularity can be studied at four different, non-exclusive levels: developmental, genetic, functional and evolutionary^20–22^. Developmental modules are defined by interactions between precursors that ultimately participate during the development of the adult structure^12^. This includes a wide range of processes, such as developmental switches leading track bifurcations of development, or signaling between tissues through different molecular mechanisms^23^. Development processes can influence each other and therefore achieve a coordinated development of tissues, organs and the whole body, promoting the so called developmental stability. Also, the relationship between development stability-instability can mediate the expression of genetic and environmental variation by transmitting their effects through different routes in development. Therefore, developmental modularity contributes to all components of phenotypic covariance between traits^24^.

Functional modularity, by contrast refers to interactions among parts of the organism performing a given function^1^. The typical example in the human face includes the array of muscles and bones that perform mastication, which generates intense strains able to modify the osseous phenotypes by, for instance, bone remodeling^1,25^.

Genetic modularity refers to patterns of joint effects between genes on traits that can be represented as a network of relationships between pleiotropic traits^26^. This type of modularity needs to be placed in the context of the “genotype-phenotype map”^1,27^. Because developmental processes mediate the expression of genetic variation in phenotypic traits, developmental and genetic modularity are related. As stated by Klingenberg^1^, this relationship need not be a perfect congruence, however, because the expression of genetic variation is not exclusively controlled by developmental interactions^1^. Moreover, genetic changes can influence developmental modularity by causing alterations in the interactions among the developmental pathways that affect the traits of interest^1,28^.

Finally, evolutionary modularity is intended as the coordinated evolutionary divergence in different traits. As genetic variation is a critical determinant for evolutionary change by selection and drift^29^, genetic parcellation contributes substantially to evolutionary modularity. Functional modularity is also an important determinant of evolutionary diversification because it provides a link between the modular structure of morphological traits and selection on performance in organismal functions^1^.

As observed, the four levels of modularity (and its counterpart, integration) are interrelated in complex ways. In many occasions, a common way to infer modularity at different levels departs from the analysis of phenotypic covariation patterns. As explained by Klingenberg^24^, identifying the modular components of a configuration of landmarks is an important task of morphometric analyses in evolutionary developmental biology. Since traits within modules are tightly correlated with each other, but relatively independent of traits in other modules, then hypotheses concerning the boundaries of modules in a landmark configuration can be tested by comparing the strength of covariation among alternative partitions of the configuration into subsets of landmarks. If a subdivision coincides with the true boundaries between modules, the correlations among subsets should be minimal^24^. Furthermore, this variational modularity concept can be applied to both, the symmetric and the asymmetric components of total shape variation, and the signals of modularity in both morphospaces can be potentially informative of modularity acting at specific levels of organization. For instance, since fluctuating asymmetry has been postulated as an indicator of developmental instability (DI), the conservation or disruption of modularity and integration patterns across several genomic backgrounds can be informative of aspects of the developmental and genetic architecture of the human face.

The metaphor of paths of development^23^ is useful for understanding the basis for the development of the covariance on morphological features. A development path denotes the set of processes that generate a trait^30^. Therefore, it is a term that incorporates molecular and cellular mechanisms underlying development processes, resulting in complex networks of interactions^30^. To characterize developmental integration/modularity it is important to distinguish the different origins of morphological covariation. A range of different mechanisms of development can produce interactions between developing traits and, therefore, generate covariation between them^1^. As Klingenberg^31^ proposed, a way to identify developmental covariation or disruption simultaneously controlling for the effects of genetic and environmental covariation is to analyze patterns of fluctuating asymmetry (FA). Because the left and right sides of an individual share the same underlying genome, the study of FA is an effective way to detect developmental noise and minimize the effects of among-individuals genetic and environmental variation. In other words, covariation in the FA of different traits is due solely to the covariation of direct development pathways^28,31,32^. Therefore, it is possible to evaluate the role of direct developmental interactions that generate covariance by comparing between versus among-individuals FA covariation patterns. Thus, by quantifying FA one can test whether patterns of developmental interactions (observed in the asymmetric component) correspond to the main components of among-individual variation, as would be expected if the integration of development is a significant constraint on evolutionary variation^33^.

**Figure 1:**
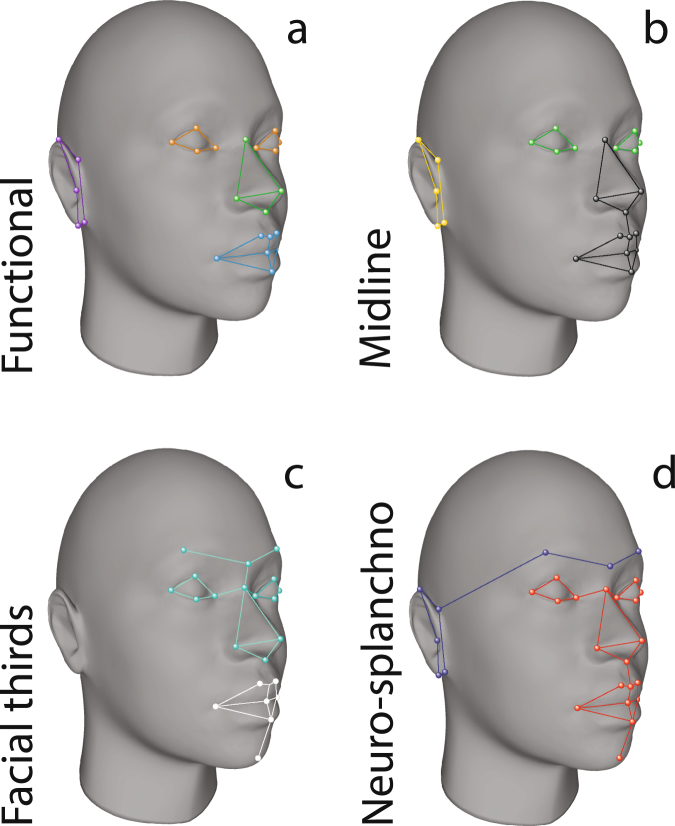
Anatomical location of the landmarks used in this study and modularity hypotheses studied here. (**a**) Functional modularity hypothesis (FMH): eyes = orange, mouth = blue, purple = ears, nose = green. (**b**) Midline modularity hypothesis (MMH): sagittal = black, midsagittal = green-olive, lateral = yellow. (**c**) Facial thirds modularity hypothesis (FTMH): middle = blue-turquoise, inferior = white. (**d**) neurocranium-splachnocranium modularity hypothesis (NSMH): neurocranium = blue-dark, splanchnocranium = red.

Here we aim to explore several modularity hypotheses in the human face, comparing their stability on the asymmetric and symmetric components of shape variation, and testing for covariation disruptions across several subsamples of Latin American mestizos differing in their Native American, European or African genomic ancestry. Specifically, we have used a composite sample of admixed Latin Americans including landmark data aimed to recover facial shape, along with genomic estimations of ancestry. Then, we have used a covariational statistical approach to test for modularity patterns in both, the symmetric and asymmetric component of facial shape variation, as a way to identify associations among developmental and among-individual variation. By organizing the original landmark configuration into several arrays, here we evaluate four general facial modularity hypotheses: a) functional modularity hypothesis (FMH), b) midline modularity hypothesis (MLMH), c) facial thirds modularity hypothesis (FTMH), and d) neurocranium-splachnocranium modularity hypothesis (NSMH) (see Fig. 1). Since we have recently reported that genetic ancestry is related to the patterns of directional and fluctuating asymmetry in the same sample of Latin-American mestizos,^34^ we replicated all the analyses on ancestry-based subsamples, to see if the recent microevolutionary history of the studied groups affect the covariation patterns, or else remain stable across the different genetic backgrounds.

To further explore the modular structure of the face, we investigated the magnitude of integration of different facial structures. Theoretically, integration is stronger if all the variation is concentrated in a single dimension of the morphospace, indicating a perfect correlation of all traits, and is absent if the variation is uniformly distributed across all available dimensions. Accordingly, morphological integration in geometric morphometric data can be measured as the scaled variance of the eigenvalues (SVE) of a principal component analysis^35^, computed on the phenotype under study. When only a few eigenvalues are large relative to the rest, then the variance of all eigenvalues will be higher than if all eigenvalues explain similar amounts of variation. In the case that the variance is large, it is considered that the trait analyzed is strongly integrated, as variation is confined to a limited morphospace in relation to the theoretical total morphospace^36^. Hallgrimsson *et al*.^14^ argued that the increase in the variance does not necessarily imply an increase in integration, and propose that to verify whether integration (as measured using SVE) is accompanied by an increase in the phenotypic variance (measured as the trace of the variance/covariance TVC) then the regression of SVE on TVC should be large and significant when the structure strongly covariates and phenotypic variance is high, as well. Thus, we complete our analyses by exploring putative disruptions in the internal integration of facial traits according to the Hallrimsson *et al*. proposal.

## Results

**Figure 2:**
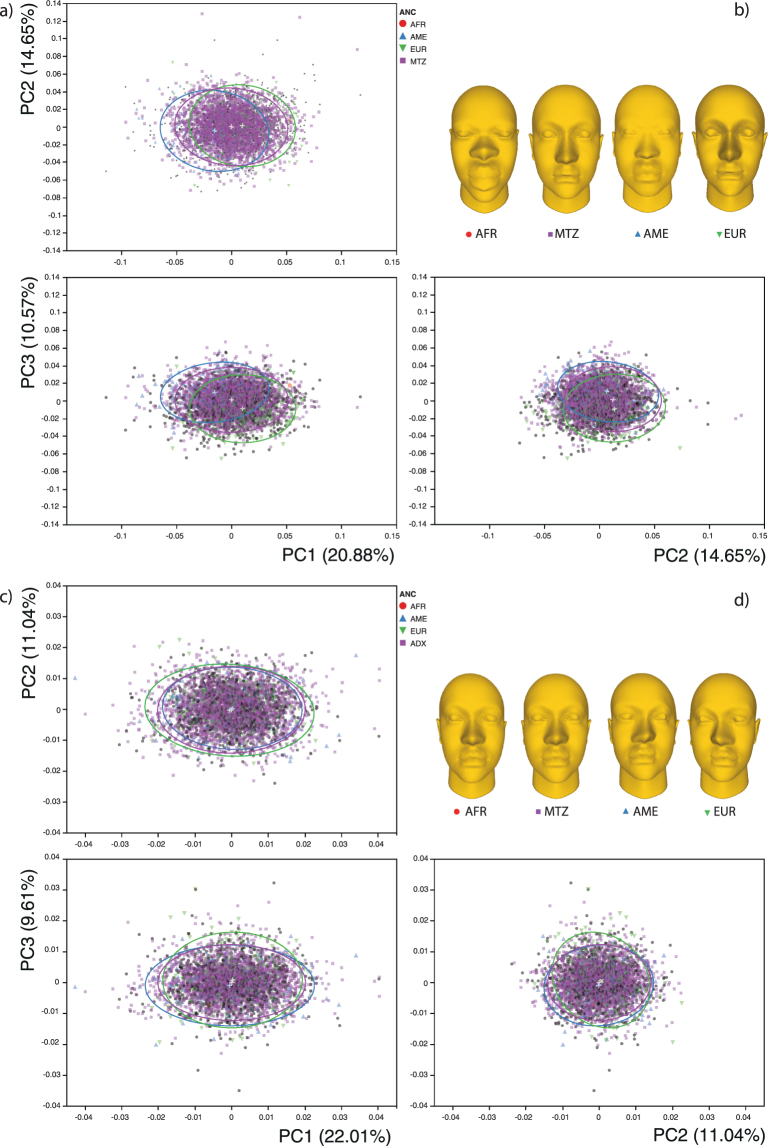
Principal Components Analysis. Scatterplots and morphings of the symmetric (**a**) and asymmetric (**c**) component of shape variation. Ellipses represent the 90% of the variance and colored by genetic ancestry (afr = red circule, ame = blue triangle, eur = green triangle, adx = purple square). (**b** and **d**) Morphed heads representing the mean shape observed on the four ancestry groups computed through discriminant analysis.

An exploratory Principal Component Analysis of the symmetric and assymetric morphospaces is presented in Fig. 2. Morphings presented in the figure depict shape variation among the centroids of each ancestry subsample. Further details concerning shape variation in this sample can be consulted in refs^34,37^. In the symmetric morphospace, the first PC show facial shape changes associated with eyes placement in relation to other structures, being more inferiorly placed in the positive scores. Also, the positive values exhibit more anteriorly and superiorly placed mouths and chins. The perifrontal region is displaced forwardly in the positive scores, whereas the ears are placed slightly higher and posteriorly in the positive values. The second PC describes shape changes related to the eversion of the ears, along with a reduction of the distance between them. The asymmetric morphospace can be decomposed on a first PC describing changes associated to directional asymmetry towards the right side in the positive scores. The eyes seem to be the more symmetric structures.

Evaluations of the hypotheses of modular organization and the magnitude of integration between the subsets of landmarks was quantified as the covariance ratio^38^. For random sets of variables, the covariation ratio (CR) has an expected value of one. While CR values lower than one will indicate some degree of modularity within the structure, CR values higher than one will indicate greater covariation between regions than within them^38^. Results corresponding to the Adams’ CR modularity index^38^ indicated that all the modularity hypothesis are significant with *p* < 0.006 (Table 1). This indicates both, that modular organization is perceived as a patent process in the human face, and that the modular hypotheses analyzed here are, to some extent, coincident in their conception.

**Table 1:** Adams’s modularity test for the total sample and subsamples, modularity hypotheses and shape components. Columns presents covariance ratio (CR) and *p*-value. Bold and italic indicate the lowest CR value for asymmetric and symmetric component of the shape variation, respectively.

Source	Module	Component	CR	P-value
Total	Functional	Asymmetric	**0**.**4841**	0.001
		Symmetric	*0*.*6148*	0.001
	Facial third	Asymmetric	0.5030	0.001
		Symmetric	0.8151	0.005
	Middle line	Asymmetric	0.5320	0.001
		Symmetric	0.6559	0.001
	Neuro-face	Asymmetric	0.7145	0.003
		Symmetric	0.7531	0.001
American	Functional	Asymmetric	**0**.**5534**	0.001
		Symmetric	*0*.*6498*	0.001
	Facial third	Asymmetric	0.5778	0.001
		Symmetric	0.8542	0.006
	Middle line	Asymmetric	0.6052	0.001
		Symmetric	0.6901	0.001
	Neuro-face	Asymmetric	0.8006	0.004
		Symmetric	0.7651	0.001
European	Functional	Asymmetric	**0**.**4997**	0.001
		Symmetric	*0*.*6110*	0.001
	Facial third	Asymmetric	0.5264	0.001
		Symmetric	0.8155	0.001
	Middle line	Asymmetric	0.5265	0.001
		Symmetric	0.6572	0.001
	Neuro-face	Asymmetric	0.6890	0.002
		Symmetric	0.7557	0.001
Admixed (Heterozygous)	Functional	Asymmetric	**0**.**4903**	0.001
		Symmetric	*0*.*6238*	0.001
	Facial third	Asymmetric	0.5039	0.001
		Symmetric	0.8388	0.005
	Middle line	Asymmetric	0.5411	0.001
		Symmetric	0.6670	0.001
	Neuro-face	Asymmetric	0.7292	0.003
		Symmetric	0.7532	0.001
Homozygous	Functional	Asymmetric	**0**.**5084**	0.001
		Symmetric	*0*.*6152*	0.001
	Facial third	Asymmetric	0.5316	0.001
		Symmetric	0.8432	0.006
	Middle line	Asymmetric	0.5440	0.001
		Symmetric	0.6561	0.001
	Neuro-face	Asymmetric	0.7286	0.003
		Symmetric	0.7692	0.001

However, specific particularities can be observed (Table 1), that inform about some modular patterns that seem to be supported by the CR coefficients as seen collectively. For instance, the “Functional” hypothesis, representing a modular organization inspired in the craneo-functional matrix theory and reflecting a key role of soft tissues in the modular formation of the face, exhibit the lowest CR values in both, the symmetric and the asymmetric components of shape, thus supporting a stronger modularity signal. Furthermore, this modularity signal is conserved across all the ancestry subsamples, suggesting that the genetic-phenotypic map intervening in the determination of adult facial phenotypes were not affected by the recent admixture dynamics.

Klingenberg and McIntyre^39^ proposed that if at given developmental process is operating on a particular morphology, the covariance matrices of the FA and individuals should be similar and proportional. The correlations between covariance matrices of FA and individual variation show, in general, a significant signal of proportionality among these matrices (Table 2). A more detailed inspection of matrix correlations performed on the different subsamples indicate some degree of variation among the proportionality of the developmental noise, depicted by the FA covariance matrix, and the among-individuals genetic/environmental covariance matrices (Table S1). For instance, modules such as the mouth and nose tend to show low, non-significant correlations among the FA and the individual matrices, suggesting a disruption among developmental pathways and the genetic/environmental basis for such traits. Conversely, regions such as the neurocranium and the eyes tend to exhibit higher than the average and significant correlations, indicating an alignment across the genetic-development-phenotype map.

**Table 2:** Values of correlation matrices of individual variation (Ind), fluctuating asymmetry (FA), and measurement error (error) for the total sample (see Table S1 for details of subsamples). Significance values obtained after 10,000 permutations of the original data. The bold show the highest correlation.

Source of variation	Iteration/module	Correlation	p value
Total sample	Ind x FA	0.4952	<0.0001
	Ind x error	**0.5288**	<0.0001
	FA x error	0.3829	<0.0001

**Figure 3:**
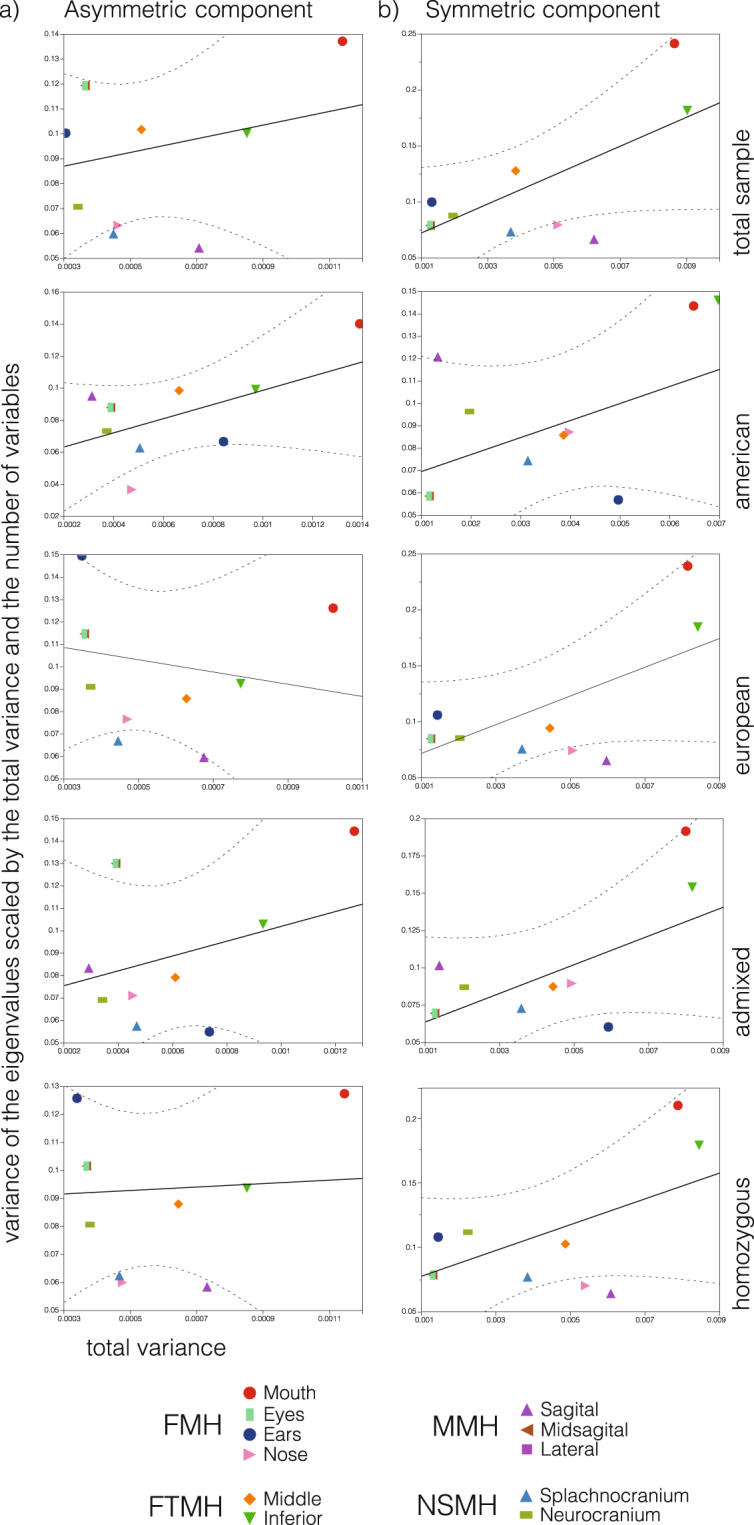
Linear regression of the variance of the eigenvectors scaled by the total variance phenotypic variance (trace), and scaled by both, the number of variables and the trace for the total sample and ancestry sub-samples. (**a**) Asymmetric component, (**b**) symmetric component. Solid black line represents the fit of the model and the dashed line curve α confidence interval 0.01. The reference for the identification of each module appears in the bottom.

Results concerning the eigenvector variance (in both versions, scaled by total variance, and scaled by total variance and number of variables, Table 3) indicate that in both, the asymmetric and symmetric components, the more integrated landmark configurations are the mouth, the eyes, the sagittal plane, and the splachnocranium (see Supplementary Fig. S1). When such proxies to integration are corrected for total variance, as recommended by Hallgrimmson *et al*., the signal of integration is limited to specific cases. In fact, the regression of morphological integration, intended here as the eigenvectors variance scaled by the total variance (Evstv) on the variance of the structure, intended here as the trace of the covariation matrix, indicates a significant relationship only for the symmetric component in the total sample and the European subsamples (see Table 4 and Fig. 3). Besides significance, there are recurrent behaviors across the different subsamples in both asymmetric and symmetric variation. Note, for instance, that the landmark configurations depicting the sagittal plane, the ears, and the splachnocranium tend to be below of the confidence limit for the regression of morphological integration on variation. This indicates that these traits exhibit greater variation than expected for their degree of internal integration, and thus suggest a lower degree of canalization of such phenotypes (see Fig. 3).

**Table 3:** Values of total variance, variance of the eigenvectors, eigenvectors variance scaled by the total variance (Evstv), and previously scaled also for the total variance and number of variables (Evstvnv). Data for the total sample are presented, see Table S2 for details on subsamples. Italic indicate morphological integration in the symmetric component, with greater values in bold and underline. The same criteria for the asymmetric component, in bold tones.

Source of variation	Hypothesis	Module	Total Variance		Eigenvalue Variance		Evstv*		Evstvnv**	
			Asymmetric	Symmetric	Asymmetric	Symmetric	Asymmetric	Symmetric	Asymmetric	Symmetric
Total sample***	Functional	Total face	0.0004	0.0031	2.20E-10	1.37E-08	0.0015	0.0015	0.0658	0.0760
		Mouth	0.0011	0.0086	2.47E-08	1.97E-06	**0**.**0191**	*0*.*0264*	0.1372	*0*.*2416*
		Ears	0.0003	0.0013	7.14E-10	1.43E-08	0.0077	0.0083	0.1003	0.0998
		Eyes	0.0004	0.0013	1.58E-09	1.38E-08	0.0118	0.0087	**0**.**1195**	0.0791
		Nose	0.0005	0.0051	2.98E-09	3.33E-07	0.0141	0.0127	0.0633	0.0792
	Middle line	Sagital	0.0007	0.0062	2.05E-09	1.43E-07	0.0041	0.0037	0.0539	0.0667
		Midsagital	0.0004	0.0013	1.58E-09	1.38E-08	0.0118	0.0087	0.1195	0.0791
		Lateral	0.0003	0.0013	7.14E-10	1.43E-08	0.0077	0.0083	0.1003	0.0998
	Facial third	Middle	0.0005	0.0039	1.84E-09	1.05E-07	0.0063	0.0071	0.1018	0.1280
		Inferior	0.0009	0.0090	8.92E-09	1.33E-06	0.0123	0.0164	0.1006	0.1819
	Neuro-Splach	Neurocranium	0.0003	0.0020	4.91E-10	1.98E-08	0.0042	0.0052	0.0709	0.0879
		Splanchnocranium	0.0005	0.0037	4.67E-10	3.14E-08	0.0023	0.0023	0.0598	0.0736

**Table 4:** Results of the regression of morphological integration on the total variance (Evstv) for symmetric and asymmetric shape components. R^2^, RMSE, df, SS, MS, F, and p values are shown. Bolded values indicates significance at α = 0.05.

Component	Effect	r^2^	RMSE	df	SS	MS	F	p
Asymmetric	Total sample	0.0700	0.0280	1	0.0005	0.0005	0.6775	0.4317
	American	0.3016	0.0236	1	0.0022	0.0022	3.8865	0.0802
	European	0.0383	0.0320	1	0.0004	0.0004	0.3586	0.5640
	Admixed/Heterozygous	0.0894	0.0331	1	0.0010	0.0010	0.8839	0.3717
	Homozygous	0.0037	0.0277	1	0.0000	0.0000	0.0337	0.8585
Symmetric	Total sample	0.4892	0.0410	1	0.0145	0.0145	8.6191	**0**.**0166**
	American	0.2203	0.0316	1	0.0025	0.0025	2.5427	0.1453
	European	0.4213	0.0432	1	0.0122	0.0122	6.5528	**0**.**0307**
	Admixed/Heterozygous	0.3579	0.0351	1	0.0062	0.0062	5.0163	0.0519
	Homozygous	0.3328	0.0405	1	0.0074	0.0074	4.4886	0.0632

## Discussion

In this paper we evaluated the most classical modularity hypothesis in order to detect if one of them fits better the observed variation in two different morphospaces: the asymmetric and the symmetric components of shape variation. Also, we have explored if the detected patterns remain stable across different subsamples differing in their genomic ancestry. Our analyses are aimed to infer the relationship among diverging environmental or genetic perturbations assumed to be promoters of phenotypic variation, and to identify the perturbations due to putative changes in the development pathways underlying the facial phenotype^40^. In this context, integration/modularity processes acting at the developmental level can suggest an important role of development as a promoter of stability or disruption of covariation patterns^33^. Finally, we intended to test for differences in the integration/variation ratio across different ancestry contexts and different facial anatomical structures, in order to corroborate if levels of developmental, genetic, or environmental signals are congruent or, conversely, show some degree of variation^28,31,32^.

So far, studies exploring modularity patterns on the external, soft facial structures are absent or scarce. Classical studies of phenotypic modularity focused primarily on the analysis of functional or developmental skeletal units^41,42^. For example, a recent study has indicated that the semi-independency of modules is not only characterized by its spatial aspects, but also for its own temporary ontogenetic structure^43^, which reinforces the recognition of the integrative and complex role of development. Modularity patterns observed in our samples confirmed the intrinsic complexity of the human face suggested in previous reports.

All the modularity hypotheses obtained statistical support, in part because they overlap in terms of landmark allotting into similar structures, but also because of the complexity of the phenotype under study. As already outlined, the differences between the symmetric and asymmetric modular patterns may be related to differential responses to gene expression during the development of the left and right sides. Some authors have hypothesized that patterns of genetic modularity and development must evolve to match the patterns of functional requirements^44–47^. If so, patterns of modularity should be equivalent in both, the asymmetric and symmetric components. In the samples analyzed here, it seems clear that modularity at the asymmetric level, reflecting patterns of covariation due exclusively to developmental factors, do not differ from the modularity pattern arising from the symmetric morphospace. Thus, a likely explanation to this observation is that developmental and genetic/environmental parcellation are somewhat aligned.

Regarding the performance of the different modularity hypotheses, in both morphospaces the functional model performs better than the remaining modularity schemes, which indicates that modularity driven by organic functioning of the craniofacial complex is observed at the developmental level and maintained at the across-individuals adult phenotypes. This suggest that the modular pattern that operates within the asymmetric and symmetric morphospace are related to function, primarily (e.g. reflecting a relative independence of the eyes, nose, mouth and ears). Such functional patterning operating at the developmental scale have been reported previously^19,48^. Our results are in accordance with previous ones^15^ in the sense that a traditional modular division into neurocranium, base and splanchnocranium^9,49^ can be more complex than previously thought. The difficulty in exploring the relationship between craniofacial modules can be discussed from the perspective of the Palimpsest model, which holds that several covariation-generating factors influence each other over time, making the inference and detection of such factors from the mere observation of adult’s phenotypes a difficult task^14^. In addition, Esteve-Altava *et al*.^50^, reported that modular patterns differ in relation to laterality, especially in the jawbone. In the same line, they define until four facial subunits with different modular variation in the sides of the face when compared to bony and muscular tissues^19^. However, note that the Esteve-Alta anatomical networks paper^12^ deals with a notion of modularity that is not related to variational modularity and thus differs from our study of phenotypic covariance patterns.

Another remarkable point is that the abovementioned results remain stable across different subsamples diverging in terms of ancestry/heterozigosity. It is interesting to note that, even when we previously reported differences in the asymmetric facial phenotypes of the different ancestry sub-samples used here^34^ the modular pattern seems to remain unaltered across all of them, reinforcing the idea that phenotypic evolution can be canalized throgouth stable patterns of covariation^51,52^. In this regard, González-José *et al*.^51^ showed that inter-population differences in the matrices of variance/covariance are not associated with matrices of molecular or morphological distances among modern human populations, suggesting that the stability of integration is independent of the history and structure of populations. They also argued that a possible explanation for this result is that patterns of integration could be limited to intraspecific level and, in the skull, some speciation events may involve large rearrangements of integration patterns that would facilitate developmental scenarios on different regions of the skull^51^. The above assumes that morphological integration of functionally or developmentally related traits will be consistent and they will respond to selective pressures on a coordinated way^44,46,53–55^.

To sum up, our results suggest that a modular organization directed by the functionally-based apportionment of covariation patterns can be seen as a regular pattern maintained across both, the genotype-phenotype map, and across several genomic backgrounds.

That the modularity/integration patterns are stable across sub-samples with different genetic ancestry is not that surprising. Previous research has already reported stability when considering different human populations (see González-José *et al*.^51^), even when individuals with a disease-altered facial development are considered. For instance, Martínez-Abadías *et al*.^56^ suggested that FGF/FGFR signaling is a covariance-generating mechanism established early in vertebrate evolution that acts as a global factor modulating coordinated development of various skull components. Thus, this as well as other unknown global genetic and non-genetic factors can be seen as covariance-generating processes that constrain both, normal or disease-altered craniofacial phenotypes Martínez-Abadías *et al*.^57^. Recent research has also clarified that the signaling activity of mutations at systems such as the FGF/FGFR are not restricted to skeletal tissues, but also occurs at cellular levels, where signaling may trigger specific patterns of gene expression and/or cell/tissue differentiation Martínez-Abadías *et al*. (2013)^58^.

Regarding the stasis/disruption of covariation patterns, Klingenberg^21,28,31^ argued that if there is a significant correlation between the covariance matrices of individual variation and fluctuating asymmetry, then it is likely that both were modeled similarly during development. Thus, any difference between the individual and FA patterns would be the result of a chronological divergence between the times in which each morphospace is modeled during development. In this context, our results suggest that the same processes are involved in the generation of the observed variation in the two levels. In other words, genetic and environmental factors that cause variation between individuals are suspected to produce patterns of change that are similar to the processes acting at the within individuals level, creating differences between the sides of the body.

An inspection of the literature on this topic appears as inconclusive regarding the congruence among the individual and the FA covariation matrices. For instance, Debat *et al*. reported a null congruence in the case of the mouse skull^56^, and suggest that it could operate some buffering against the impact of different sources of variation operating on distant regions such as the nasal capsule, the zygomatic arch, the orbit, and the cranial vault, etc. Similarly, Klingenberg *et al*.^59^ found no congruence between individual covariance matrices and FA in pharyngeal jaws of cichlid fishes, which is associated with a polymorphism identified as a source of discrepancy. Similar tests performed on insects, however, report congruence among both covariation matrices (see refs^39,60^ for flies, and ref.^61^ for bumblebees). Several factors can be listed to explain these differences. One is the obvious discrepancy between genetic and developmental pathways in insects, mice, fish and humans. Phylogenetic analyses at the macro-evolutionary level are then necessary to answer these questions on an evolutionary context. Moreover, Debat *et al*.^56^ suggest that natural selection acting on the shape of the wings of insects could dilute or hide the differences between within and among-individuals developmental processes. In the case of the human face, selection processes have also been postulated^62,63^, thus part of its expression pattern could be mediated by past evolutionary events.

A more detailed inspection of the discrepancies among FA and individual covariance matrices (Table S1) enabled us to investigate more fine differences among covariance patterns. Note, for instance, that the eyes, ears and neurocranium modules would be affected by parallel or equivalent noise-stability development processes. The complexity of the mosaic expression of developmental pathways is suggested indirectly on such results: the eye region develops around day 22, with the appearance of a pair of shallow grooves on both sides of the forebrain^64^. In addition, the medial migration of the eyes from their initial side locations results on the massive growth of the cerebral hemispheres and enlargement of the head, and enable the normal movement of eyes toward the sagittal line^65^. The largest migratory eye movements occur between the fifth and ninth week of the development. Thereafter, the angulation of the optical axes (between 71°–68°) is stabilized during postnatal phases^65^. The genetic basis of regulation of the eye is well known and is associated with PAX6^64^, with intervention of the sonic hedgehog (SHH) to the up regulation of PAX2 and down regulation of PAX6. Moreover, fibroblast growth factors (FGF), and transcription factors MITF, CHX10, SOX2, BMP-4, LMAF, SIX3 and PROX1 determine the course of ocular substructures.

Regarding ears’ development, they begin its formation around day 22 and its development extends to week six. Specifically, their tissues derive from ectoderm precursors. The pavilion and the ear meatus move from the base of the neck (cervical region) to its normal adult location on the side of the head. This process is largely due to mandibular growth. On a recent GWAS analysis, we demonstrated that there are seven genomic regions significantly associated with the size and the attachment of the lobe, folding of antihelix, rotation of the helix, the protrusion of the ear and the antitragus size^66^. These features are associated with variants of EDAR, TBX15 and CART1 genes. In summary, the parallelism between individual and FA covariation patterns seems to be supported by the synchronic timing of developmental events in the growing embryo, and some candidate genes have been detected whose role must be further investigated.

In contrast, the mouth and the nose arise from a rather complex combination of developmental processes, i.e.: they condense the combined action of several mechanisms, tissue origins and differentiation types. Notably, they come from the same layer, the endoderm, forming during the middle of week three and keeping their development until birth. During the half of the sixth week only the oronasal membrane divides the oral and nasal cavities. The mouth results from the interrelation of the maxillary and mandibular prominence, and besides depends on the closure of the palate, the vomeronasal system, the configuration of the nose and in general of all the pharyngeal cavity^64^. The complex processes described above may explain the discrepancy (or lack of proportionality) of the covariance matrices. In another recent GWAS study, four genomic regions (4q31, 6p21, 7p13, and 20p11) were significantly associated to features related to the nose morphology: the inclination of the columella, the amplitude of the nasal bridge, and the alar amplitude of the nose (with p-values = 3 × 10.9 to 9 × 10-9). The reported SNPs are associated to DCHS2, RUNX2 and GLI3 genes, respectively, while the region 20p11 overlaps with PAX1^67^. Thus, the hits in different and non-overlapping genetic systems also correlates with different phenotypes: the ear structures nucleated in the pinna^66^, versus localized nasal traits^67^. In some way, these combinated results can be seen as a preliminary signal of genetic modularity, detected by different methods as the ones used here.

Note that mouth and nose, as already mentioned, are structures where several functional, developmental, and growth processes simultaneously converge. Maybe, this complexity trigger the observed differences in the covariance patterns observed on eyes, ears and neurocranium. A point to note is that all the comparisons made in the middle third, which include three landmarks associated with the neurocranium, nose, and mouth (Figs 1 and 3) were statistically significant. This may endorse the reported influence of neurocranium in shaping splachnocranium^49^.

Moreover, natural selection acting quite independently upon the modules^56^, the complexity of structures^68^, and the phenotypic plasticity of the nose and mouth, as a whole, can be a plausible explanation to the observed differences in terms of matrix proportionality. For example, the maxillary region exhibits large craniofacial plasticity^69^, maybe due to the fact that the masticatory apparatus grows even up to 25 years^65,70^. Similarly, in the case of the nose, there is evidence of plasticity associated with the climate and it is possibly a structure that evolved under a regime of natural selection to cold climates^71–73^. Considering all the above, it is likely that the mouth and nose do not achieve the integration levels exhibited by other facial modules.

Most, if not all evidence provided so far by previous studies^15,51,52,56,74–76^ focuses on the symmetric component form. Instead, our approach was twofold, focusing also in the asymmetric dimension of variation. In this regard, a noticeable result is that the eyes, mouth, and ears tend to present larger-than-average morphological integration (Table 3). Previous asymmetry studies suggested that integration at the development level can play an important role in canalization of variation through periods of environmental change. Such statements seems to be supported by a study of skulls of Late Pleistocene carnivores and the relationship between fluctuating asymmetry, phenotypic integration, variance and environmental change or stress^33^, and similar results are corroborated in mandibles of shrews (for details see refs^77,78^). In the abovementioned examples, the effects of environmental stress artificially induced the integration of development and variation in the jaws of shrew, and also showed that fluctuating asymmetry and the variation of the stressed population increased and that this increasing was canalized along the same direction of between-species variation^77,78^. In this context, we have obtained a recurrent correlation between the pattern of fluctuating asymmetry and morphological integration across all the subsamples in the symmetric component of shape, which is not the case when the asymmetric morphospace is explored for such correlation (Table 5). In this sense the regression trends corresponding to each genomic subsample are roughly equivalent (i.e. are positive), excepting for the European descent sample, that shows a pattern of negative correlation between asymmetry and integration in the asymmetric morphospace (Table 5). This suggest that the relationship between asymmetry and integration differs at the symmetric and asymmetric morphospaces. Theoretically, we could infer that the developmental pathways operating on individuals of (mostly) European-descent are divergent, to some extent, a statement that supported also by their differential expression on the pattern of facial asymmetries that we have reported recently^34^. Since functional structures are expressed early during prenatal development, it is expected that developmental modularity can potentially influence their functional counterpart, although more research is needed to disentangle the robustness of the relationship between these two modularity types^79^. Of course, the inverse explanation cannot be discarded, and it could be the case that functional modularity influences developmental processes such as bone remodeling and other forms of plasticity in which the loading mechanisms influence the rates and direction of tissue growth^80,81^, thus promoting modularity at the developmental level. While previous analyses have hypothesized that selection makes functional and genetic modularity converge^22,44,45,82,83^, other studies indicate that different morphological structures can perform equivalent functions and, therefore, there can be considerable flexibility in the neutral divergence^84,85^ or among-trait connectivity, independent of their function^19,48^. Whatever the case, our results indicate that the modular landscape of the human face can be affected by both, genetic ancestry and the morphospace under study (asymmetric versus symmetric). Furthermore, these results are of importance to future Genome Wide Association Studies based on CANDELA (and other) datasets, since they provide a first identification of integration and modularity at the phenotypic scale, that can be used to submit more refined traits to the association analyses, as a preferred approach instead of heuristic searches based on analyzing non-supervised, raw characters.

**Table 5:** Regression results of fluctuating asymmetry (FA) on morphological integration (Evstv) in both components of the shape, for the total sample and subsamples (Native American, European, Admixed/Heterozygous, Homozygous). R^2^, root mean square error, correlation, degree of freedom, sum squares, mean squares, F value and p value are presented. The bold represent the unique test with statistical significance with α = 0.05. Italic indicate the higher correlations.

Component	Effect	r^2^	RMSE	Correlation	df	SS	MS	F	p
Asymmetric	Total sample	0.0709	0.0280	0.2662	1	0.0005	0.0005	0.6864	0.4288
	American	0.2011	0.0252	0.4484	1	0.0014	0.0014	2.2649	0.1666
	European	0.0051	0.0326	−0.0715	1	0.0000	0.0000	0.0463	0.8344
	Admixed/Heterozygous	0.2757	0.0295	*0*.*5251*	1	0.0030	0.0030	3.4257	0.0972
	Homozygous	0.0021	0.0277	0.0457	1	0.0000	0.0000	0.0189	0.8938
Symettric	Total sample	0.5104	0.0401	0.7145	1	0.0151	0.0151	9.3839	**0**.**0135**
	American	0.5407	0.0242	0.7353	1	0.0062	0.0062	10.5963	**0**.**0099**
	European	0.4043	0.0438	0.6359	1	0.0117	0.0117	6.1087	**0**.**0355**
	Admixed/Heterozygous	0.7377	0.0224	*0*.*8589*	1	0.0127	0.0127	25.3056	**0**.**0007**
	Homozygous	0.3437	0.0401	0.5862	1	0.0076	0.0076	4.7125	**0**.**0580**

## Materials and Methods

### The sample

As part of the CANDELA initiative, we recruited 4,104 volunteers from six Latin-American cities: Mexico City (Mexico), Medellin (Colombia), Lima (Perú), Arica (Chile), Porto Alegre, and Jequié (Brazil). The CANDELA consortium aims to evaluate the genetic basis of nonpathological phenotypes differentiated between European, American, and African populations through the analysis of admixed populations (see ref.^37^). Volunteers with antecedents of craniofacial dysmorphologies, orthodontics treatments or severe facial trauma were not considered in this study. Further sample details are provided in Supplementary Table S3. Approvals provided by the Ethics Committees of the Universidad Nacional Autónoma de México and Escuela Nacional de Antropología e Historia (México), Universidad de Antioquia (Colombia), Universidad Peruana Cayetano Heredia (Perú), Universidad de Tarapacá (Chile), Universidade Federal do Rio Grande do Sul/Universidade Estadual do Sudoeste da Bahia (Brazil), and University College London (UK) were obtained prior the data collection, and an informed consent were signed by each participant before genetic, socioeconomic, and facial phenotypes data was collected. The same sample was used on a recent contribution^34,37,86^ and conforms the main database of the CANDELA consortium^34,37,66,67,86,87^. All methods and procedures used here were performed in accordance with relevant guidelines and regulations (see below). Validation dataset are available to download from https://laofunam.com/data.

Facial shape was captured following scientific photographic protocols described in detail in references^34^ and^86^. Upon these images, two observers (MQS and LC) placed a set of 34 standard facial landmarks using Photomodeler (http://www.photomodeler.com/ Eos Systems Inc, Vancouver, Canada). As described elsewhere^34,86^, this platform corrects for any lens distortion automatically, and we have followed the standard recommendations for quality and accuracy provided by the software. A scale factor was assessed using the nasion-gnathion distance measured directly on the individuals using a standard anthropometric caliper.

On each individual, blood samples were collected and DNA extraction was performed following standard laboratory procedures. Genomic data involving 730,525 marker SNPs was obtained from these samples (see further details in ref.^37^). The SNPs were pruned to remove Linkage Disequilibrium, and after removing correlated SNPs, 90,000 SNPs were left for analysis. Ancestry estimation was performed with this SNP data. Genome-wide average heterozygosity was estimated from this data using PLiNK^88,89^, which provides a measure of excess heterozygosity compared to the overall sample. It is calculated as 1—excess homozygosity, while excess homozygosity is estimated using the inbreeding coefficient as the average excess of homozygous alleles across all SNPs for an individual as compared to the overall sample. To enable among-subsample comparisons, individuals were allotted to American, European, or Admixed groups if their population-specific ancestry markers were above 80% in the first two cases, and below that percentages in the third group (admixed).

### Preliminary statistical analyses: error measurement and acquisition of the symmetric and asymmetric components of variation

Since on a previous paper^86^ we have demonstrated that measurement error in size and shape was significantly lower than variation in FA, and thus negligible, the rest of the analyses were based on a single digitization of landmarks.

For the multilevel analysis of morphological integration and modularity, we first decomposed the total shape variation into its symmetric and asymmetric components by conducting a Procrustes ANOVA on the total dataset. The symmetric component is the variation between individuals in terms of the averages of the left and right configurations and corresponds to the phenotypic variation^59^. Conversely, the asymmetric component is the variation within individuals in terms of differences between configurations from the left and the right sides of each individual and depicts variation arising from direct developmental interactions^59^. Main shape changes in the symmetric and asymmetric morphospaces were preliminary explored using Principal Component Analysis and morphing of a scanned face to depict shape variation among the centroid of the of the genetic ancestry subsamples, via Discriminant Function Analysis.

### Modularity hypotheses

The FMH (Fig. 1a) is based on the craneo-functional matrix theory, and is aimed to represent a key role of soft tissues in the modular formation of the face which would facilitate its stability, performance and evolution^90,91^. Moss and Young^91^ argued that the head is divided into functional components (e.g. modules) determined by the soft tissues and cranial cavities, and limited by their bony surroundings. In this regard, the soft tissue components would guide the development of skeletal units. It should be noted that the functional components are based on assumptions around form and function links rather than the results of quantitative analyses or empirical evidences^19^.

MLMH (Fig. 1b) is aimed to explore if the farther a module is of the sagittal midline, the more asymmetry will display^92^. Recently, it has been suggested that the expression of FA not only depends on developmental stability, but also on the cost of growth of the trait, defined as the amount of structural components necessary to form a unit of length of a given character^68^. In accordance with this argument, a trait with more structural components per unit of length should show lower asymmetry than a simpler one. In other words, this hypothesis seeks to verify whether those landmarks that are located closer to the sagittal plane versus those placed on the sides follow a modular pattern.

The FTMH (Fig. 1c) is aimed to test a traditional classification of facial anatomy, mainly used in medicine studies^93–95^. It is important to check how much these regions respond to a modularity pattern with empirical support.

Finally, the NSMH (Fig. 1d) is an attempt to verify if the differences in development timing, tissular precursors, and epigenetic stimuli that the splachnocranium (face) and the neurocranium^41^ experience are enough to generate a modularity pattern observed in the soft structures studied here.

All the above-mentioned hypotheses will be tested in the whole sample as well as in sub-samples organized according to their genomic ancestry. Also, the hypotheses are evaluated in both the symmetric and the asymmetric components of shape variation.

The CR coefficient is a ratio of the covariation between modules over the covariation within modules, and consequently it ranges between zero and positive values^38^. For random sets of variables, the CR has an expected value of one. Whereas CR values lower than indicate some degree of modularity within the structure, CR values higher than one depict greater covariation between regions than within them^38^. Thus, we calculated the CR values on the several modularity hypotheses tested here, and across the different ancestry subsamples. The significance values were obtained by comparing the observed CR values with permutational distributions of 999 CR values obtained by assigning the landmarks randomly to modules. The proportion of permuted values lower than the observed CR value was used as the significance of the test^38^.

### Covariance matrix similarity: exploring developmental pathways

Similarity between FA and individuals covariance matrices was tested following Klingenberg and McIntyre^39^. Similarity of the covariance matrices resulting from the Procrustes ANOVA was tested by computing pairwise matrix correlations, as a way to compare FA versus individual covariance patterns. Matrix correlation is a measure of the overall similarity of covariance matrices and its use is ubiquitous in geometric morphometrics^96,97^. Statistical significances were determined through matrix permutation tests, with 10,000 iterations, against the null hypothesis of complete dissimilarity between the covariance matrices concerned^39^.

### Facial morphological integration

Theoretically, integration is stronger if all the variation is concentrated in a single dimension, indicating a perfect correlation of all traits, and is absent if the variation is uniformly distributed across all available dimensions. Accordingly, morphological integration in geometric morphometric data can be measured as the scaled variance of the eigenvalues (SVE) of a principal component analysis^35^, made on the structure under study. When only a few eigenvalues are large relative to the rest, then the variance of all eigenvalues will be higher than if all eigenvalues explain similar amounts of variation. In the case that the variance is large, it is considered that the trait analyzed is strongly integrated, as variation is confined to a limited morphospace in relation to the theoretical total morphospace^36^. Hallgrimsson *et al*.^14^ argued that the increase in the variance does not necessarily imply an increase in integration, and propose that to verify whether integration (as measured using SVE) is accompanied by an increase in the phenotypic variance (measured as the trace of the variance/covariance TVC) then the regression of SVE on TVC should be large and significant when the structure strongly covariates and phenotypic variance is high, as well. Analyses were performed on both, the symmetric and asymmetric component^39^.

## Electronic supplementary material

Supplementary information
