# Supplementary material for: Developmental pathways inferred from modularity, morphological integration and fluctuating asymmetry patterns in the human face
Source: Sci Rep. 2018 Jan 17;8:963. doi: 10.1038/s41598-018-19324-y (PMC5772513; doi:10.1038/s41598-018-19324-y)
Supplement: Supplementary file 1 — Supplementary information [file 41598_2018_19324_MOESM1_ESM.doc]

Developmental pathways inferred from modularity, morphological integration and fluctuating asymmetry patterns in the human face.

Mirsha Quinto-Sánchez^1,2^, Francesc Muñoz-Muñoz^3^, Jorge Gomez-Valdés^4^, Celia Cintas^2^, Pablo Navarro^2^, Caio Cesar Silva de Cerqueira^5^, Carolina Paschetta^2^, Soledad de Azevedo^2^, Virginia Ramallo^2^, Victor Acuña-Alonzo^6,7^, Kaustubh Adhikari^6^, Macarena Fuentes-Guajardo^6,8^, Tábita Hünemeier^9^, Paola Everardo^7,10^, Francisco de Avila^7^, Carla Jaramillo^11^, Williams Arias^11^, Carla Gallo^12^, Giovani Poletti^12^, Gabriel Bedoya^11^, Maria Cátira Bortolini^13^, Samuel Canizales-Quinteros^14^, Francisco Rothhammer^15^, Javier Rosique ^16^, Andres Ruiz-Linares^6,17,18^ and Rolando Gonzalez-José^2^,*

^1^ Ciencia Forense, Facultad de Medicina, Universidad Nacional Autónoma de México, Ciudad de México.

^2^ Instituto Patagónico de Ciencias Sociales y Humanas. Centro Nacional Patagónico, CONICET, Puerto Madryn, Argentina.

^3^ Departament de Biologia Animal, de Biologia Vegetal i d’Ecologia, Facultat de Biociències, Universitat Autònoma de Barcelona, Avinguda de l’Eix Central, Ediﬁci C, E-08193 Bellaterra (Cerdanyola del Vallès), Spain.

^4^ Posgrado en Antropología Física, Escuela Nacional de Antropología e Historia, Ciudad de México.

^5^ Superintendência da Polícia Técnico-Científica do Estado de São Paulo. Equipe de Perícias Criminalísticas de Ourinhos, São Paulo, Brazil.

^6^ Department of Genetics, Evolution and Environment, and UCL Genetics Institute, University College London, London, UK.

^7^ Licenciatura en Antropología Física, Escuela Nacional de Antropología e Historia, Ciudad de México.

^8^ Departamento de Tecnología Médica, Facultad de Ciencias de la Salud, Universidad de Tarapacá, Arica, Chile.

^9^ Departamento de Genética e Biologia Evolutiva, Instituto de Biociências, Universidade de São Paulo.

^10^ Posgrado en Antropología, Instituto de Investigaciones Antropológicas, Universidad Nacional Autónoma de México, Ciudad de México.

^11^ Universidad de Antioquia, Medellín, Colombia.

^12^ Laboratorios de Investigación y Desarrollo, Facultad de Ciencias y Filosofía, Universidad Peruana Cayetano Heredia, Lima, Perú.

^13^ Departamento de Genética, Instituto de Biociências, Universidade Federal do Rio Grande do Sul, Porto Alegre, Brazil.

^14^ Facultad de Química, UNAM, Mexico City, México.

^15^ Facultad Instituto de Alta Investigación Universidad de Tarapacá, Programa de Genética Humana ICBM Facultad de Medicina Universidad de Chile y Centro de Investigaciones del Hombre en el Desierto, Arica, Chile.

^16^ Departamento de Antropología. Facultad de Ciencias Sociales y Humanas. Universidad de Antioquia, Medellín, Colombia.

^17^ MOE Key Laboratory of Contemporary Anthropology, Fudan University, Shanghai, China

^18^ Aix Marseille Univ, CNRS, EFS, ADES, Marseille, France

Supplementary information (Figures and Tables)

Figure S1: Graph contrasting patterns of individual variation (Ind), directional asymmetry (DA), fluctuating asymmetry (FA) and morphological integration (MI) for symmetric and asymmetric shape components. Data are sorted by modularity hypothesis for the total sample and sub-samples. The lines between the values of each source of variation try to generalize the pattern within each hypothesis of modularity.


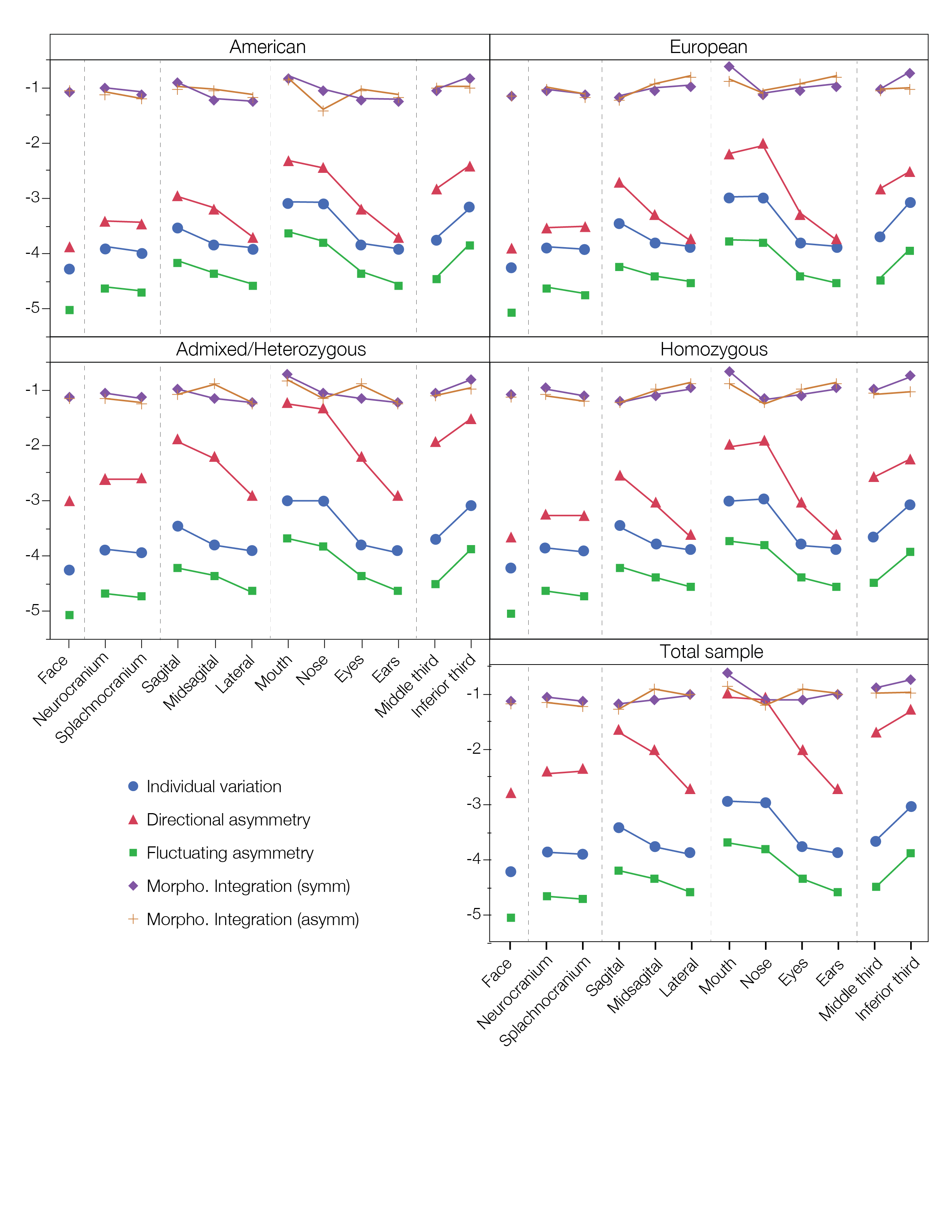


Table S1: Correlation values between matrix of individual variation (Ind), fluctuating asymmetry (FA) and measurement error for the subsamples. Significance values obtained after 10,000 permutations. The blue boxes show the highest correlation values, whereas green indicates significant correlations.

note: double click for excel table.

Table S2: Values of total variance, variance of the eigenvalues, eigenvalues variance scaled by the total variance and previous scaled also for the number of variance. Data for the subsamples (European ancestry, Native American, mestizo / heterozygous, and homozygous) are presented. Green boxes indicate greatest (darker) or intermediate (light) integration values in the symmetric component. The same criteria was applied to the asymmetric space, using a red gradient.

note: double click for excel table.

Table S3. Sample details concerning age, sex and country for sample of 4104 volunteers (see^51^).
